# Phase Variable O Antigen Biosynthetic Genes Control Expression of the Major Protective Antigen and Bacteriophage Receptor in *Vibrio cholerae* O1

**DOI:** 10.1371/journal.ppat.1002917

**Published:** 2012-09-13

**Authors:** Kimberley D. Seed, Shah M. Faruque, John J. Mekalanos, Stephen B. Calderwood, Firdausi Qadri, Andrew Camilli

**Affiliations:** 1 Howard Hughes Medical Institute and Department of Molecular Biology and Microbiology, Tufts University School of Medicine, Boston, Massachusetts, United States of America; 2 Molecular Genetics Laboratory, International Centre for Diarrhoeal Disease Research, Bangladesh, Dhaka, Bangladesh; 3 Department of Microbiology and Molecular Genetics, Harvard Medical School, Boston, Massachusetts, United States of America; 4 Division of Infectious Diseases, Massachusetts General Hospital, and Harvard Medical School, Boston, Massachusetts, United States of America; 5 Centre for Vaccine Sciences, International Centre for Diarrhoeal Disease Research, Bangladesh, Dhaka, Bangladesh; Northwestern University, Feinberg School of Medicine, United States of America

## Abstract

The *Vibrio cholerae* lipopolysaccharide O1 antigen is a major target of bacteriophages and the human immune system and is of critical importance for vaccine design. We used an O1-specific lytic bacteriophage as a tool to probe the capacity of *V. cholerae* to alter its O1 antigen and identified a novel mechanism by which this organism can modulate O antigen expression and exhibit intra-strain heterogeneity. We identified two phase variable genes required for O1 antigen biosynthesis, *manA* and *wbeL*. *manA* resides outside of the previously recognized O1 antigen biosynthetic locus, and encodes for a phosphomannose isomerase critical for the initial step in O1 antigen biosynthesis. We determined that *manA* and *wbeL* phase variants are attenuated for virulence, providing functional evidence to further support the critical role of the O1 antigen for infectivity. We provide the first report of phase variation modulating O1 antigen expression in *V. cholerae*, and show that the maintenance of these phase variable loci is an important means by which this facultative pathogen can generate the diverse subpopulations of cells needed for infecting the host intestinal tract and for escaping predation by an O1-specific phage.

## Author Summary

The O1 serogroup of *Vibrio cholerae* is the most common cause of the potentially fatal diarrheal disease cholera, which remains a significant global health burden worldwide. The O1 antigen is a constituent of the lipopolysaccharide portion of the outer membrane, and its location on the bacterial surface makes it a major target of both the immune system and bacteriophages. We used an O1-specific bacteriophage as a tool to understand if, and how, *V. cholerae* can alter O1 antigen expression. We discovered that two genes, which are critical for O1 antigen biosynthesis, are subject to phase variation. Additionally, one of the phase variable genes we identified was not previously known to play a role in O1 antigen biosynthesis in *V. cholerae*. Phase variation is a well-recognized mechanism many other bacterial pathogens use to generate variable expression of surface components, and this is generally thought to help these organisms evade the immune system. Phase variation has not previously been described as a widespread mechanism used by *V. cholerae*, furthermore, this is the first report that *V. cholerae* O1 is capable of generating diverse populations with variable and unique O1 antigen expression.

## Introduction

Lipopolysaccharide (LPS) is a prominent constituent of the outer membrane of Gram-negative bacteria. The LPS molecule is divided into three components; lipid A, core oligosaccharide and O-specific polysaccharide (or O antigen). The structure of the O antigen typically defines the serogroup of an organism, and over 200 serogroups of *Vibrio cholerae* are currently recognized [1]. Interestingly, the O1 serogroup has been and continues to be the dominant cause of both endemic and epidemic cholera throughout the world, though the reasons for this are unknown. The incidence of cholera worldwide is steadily increasing, and the cumulative number of reported cases in 2010 was nearly double what it was in 2009 [2]. When considering the level of gross under-reporting, the actual global disease burden is estimated to be 3–5 million cases and more than 100,000 deaths [3], [4]. The observed increase in reported cases in 2010 is largely due to a recent outbreak of an O1 strain that started in Haiti: Even more concerning is the observation that 53% of the global total of the number of reported deaths from cholera in 2010 occurred in Haiti in a period of only 70 days [2]. These observations highlight the fragile nature of impoverished and tragedy-struck nations to the rapid onset of cholera epidemics.

**Figure 1:**
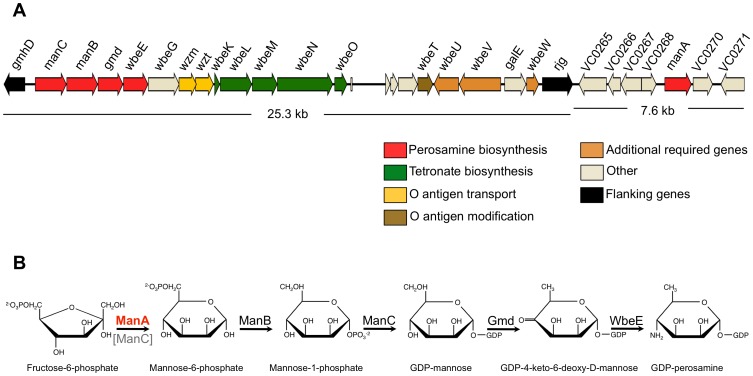
Genetic organization of the O1 antigen biosynthetic locus and the proposed pathway for the synthesis of perosamine in *V. cholerae* O1. A) Pictorial representation of the genes in the O1 antigen biosynthetic (*wbe*) locus. The annotated ORFs and their orientation are indicated by block arrows (drawn to scale). The genes currently described as being required for O1 antigen biosynthesis are located between the *gmh*D and *rjg* flanking genes. Work presented here however extends the O1 antigen biosynthetic cluster to include VC0269 (*manA*). The locus can be divided up into five major groups according to putative function that are indicated by the same color arrow. B) Proposed biosynthetic pathway for perosamine in *V. cholerae* O1. The pathway includes ManA for the conversion of F6P to M6P and eliminates ManC (indicated in grey) as the enzyme responsible for the initial step of perosamine biosynthesis.

The *V. cholerae* O1 antigen is composed of 12–18 repeating units of α(1,2)-linked d-perosamine (4-amino-4,6-dideoxy d-mannose) residues, the amino groups of which are acylated with tetronate (3-deoxy-l-glycero-tetronic acid) (Fig. S1) [1], [5]–[8]. The genes currently described as being required for the synthesis of the O1 antigen are located on chromosome 1 of the *V. cholerae* O1 N16961 genome between open reading frames (ORFs) VC0240 (*gmh*D) and VC0264 (*rjg*) (Fig. 1A) [9]. This region (the *wbe* or *rfb* region) was originally identified through the heterologous expression of the *V. cholerae* O1 antigen in *Escherichia coli* K-12 [10]. Additional genes required for the synthesis of the O1 antigen in *V. cholerae* were subsequently identified [11], however all O1 antigen biosynthetic genes studied to date have been between the *gmhD* and *rjg* flanking genes. The genes responsible for O1 antigen biosynthesis have been placed into the following five groups according to putative function: perosamine biosynthesis (VC0241–VC0244) [12]; O antigen transport (VC0246–VC0247) [13]; tetronate biosynthesis (VC0248–VC0252) [14]; O antigen modification (VC0258) [15], [16]; and additional genes essential for O antigen biosynthesis (VC0259–VC0260, VC0263) [11] (Fig. 1A). A putative pathway for the biosynthesis of perosamine has been proposed by Stroeher *et al.* [12] (Fig. 1B). In this pathway, which is based solely on homology comparisons, the first step is the conversion of fructose-6-phosphate (F6P) to mannose-6-phosphate (M6P) by ManC (a predicted type II phosphomannose isomerase [PMI]). M6P is then converted to mannose-1-phosphate (M1P) by ManB, and then to GDP-mannose by ManC. GDP-mannose is converted to GDP-4-keto-6 deoxymannose by WbeD and then GDP-perosamine by WbeE. PMIs (E.C. 5.3.1.8) catalyze the reversible isomerization of M6P to F6P and are divided into three families on the basis of amino acid sequence [17]. Type I PMIs are monofunctional enzymes and include proteins from humans to bacteria including *E. coli* [18] and *Salmonella enterica* serovar Typhimurium [19]. Type II enzymes are bacterial bifunctional enzymes possessing both PMI and guanosine diphospho-d-mannose pyrophosphorylase (GMP) activity (for the conversion of M1P into GDP-mannose) in distinct catalytic domains [20]. PMIs play critical roles in mannose catabolism and in the supply of GDP-mannose, which is necessary for the mannosylation of various structures including LPS.

The distal location of the O antigen extending outward from the bacterial surface positions it at the interface between the bacterium and its environment. As such, the O antigen is important for protection from various environmental stresses including antibiotics and the host immune response [21], [22]. The O antigen is also consequently the target of both the immune system and bacteriophages, which can independently apply powerful selective forces. As such, cell surface structures, including the O antigen, are frequently observed to exhibit high levels of variation [23]. Examples of phase variable surface structures are abundant in bacterial pathogens and include *Haemophilus influenzae* lipooligosaccharide (LOS) [24]–[26], *Neiserria meningitidis* LOS [27], *Helicobacter pylori* LPS [28], [29] and *Campylobacter jejuni* LOS [30], [31]. The loci responsible for phase variable expression of these structures, often referred to as contingency loci, are thought to offer a preemptive strategy to increase diversity necessary for bacterial adaptation in unpredictable environments [32]. Phase variation can be mediated by DNA polymerase slipped-strand mispairing across simple sequence repeats, and when located in coding sequences, can lead to a frameshift mutation resulting in the production of truncated, often nonfunctional, peptide. Homopolymeric nucleotide tracts are one subset of simple sequence repeats commonly observed to undergo frequent expansion and contraction resulting in reversible heritable phenotypic variation [23], [32].

One variation of the *V. cholerae* O1 antigen that has been demonstrated and which defines the two serotypes, Ogawa and Inaba, is the presence or absence, respectively, of a terminal methyl group [16]. The two serotypes can undergo serotype conversion during epidemics or in endemic areas [33]–[38]. Spontaneous mutations in the predicted methylase *wbeT* (VC0258) are linked to this switching phenotype [15] and may be involved in immune evasion as cross-serotype protection is limited. In contrast to the immune pressure being somewhat specific for a given serotype, bacteriophages that target the O1 antigen for use as a receptor may not be serotype-specific. In this regard, it has recently been reported that the absorption of several different O1-specific phages to *V. cholerae* can be modulated by its cyclic AMP (cAMP)-cAMP receptor protein regulatory system, suggesting that regulatory pathways may exist that alter O1 antigen abundance or surface organization [39]. We recently described ICP1, an O1-specific, but serotype nonspecific *V. cholerae* phage that is prevalent in cholera patient stool samples in the cholera endemic region of Bangladesh [40]. ICP1 is likely related to the previously described phage JSF4 [41]. We sought to investigate the mechanisms employed by pathogenic *V. cholerae* O1 to resist ICP1 infection and discovered two phase variation mechanisms by which *V. cholerae* O1 displays intra-strain O antigen heterogeneity. This heterogeneity is mediated by two contingency loci involved in tetronate biosynthesis (*wbeL*) and a previously unrecognized PMI (*manA*) critical for perosamine biosynthesis.

## Results/Discussion

### Identification of contingency loci controlling *V. cholerae* O1 antigen biosynthesis

Plaques resulting from the infection of a wild type *V. cholerae* O1 strain with the O1-specific phage ICP1 were routinely observed to have colonies growing in the center indicating the presence of phage resistant isolates. Four independent phage resistant isolates were subjected to whole genome resequencing and the majority of the isolates (three of four) had single nucleotide deletions that mapped to homonucleotide (poly-A) tracts within two genes. Two mutants were found to have a deletion in the poly-A (A_8_) tract starting at nucleotide position 108 in *wbeL*, a gene that is predicted to be required for tetronate synthesis [14]. The full-length WbeL protein is 471 amino acids and a single nucleotide deletion within the poly-A tract results in the production of a truncated peptide of 42 amino acids due to a premature stop codon 12 nucleotides downstream of the poly-A tract (Fig. S2). *wbeL* is unique to *V. cholerae* O1 strains, and the poly-A tract is 100% conserved in all 37 *V. cholerae* O1 strains available for analysis through the National Center for Biotechnology Information (NCBI) DNA sequence database.

**Figure 2:**
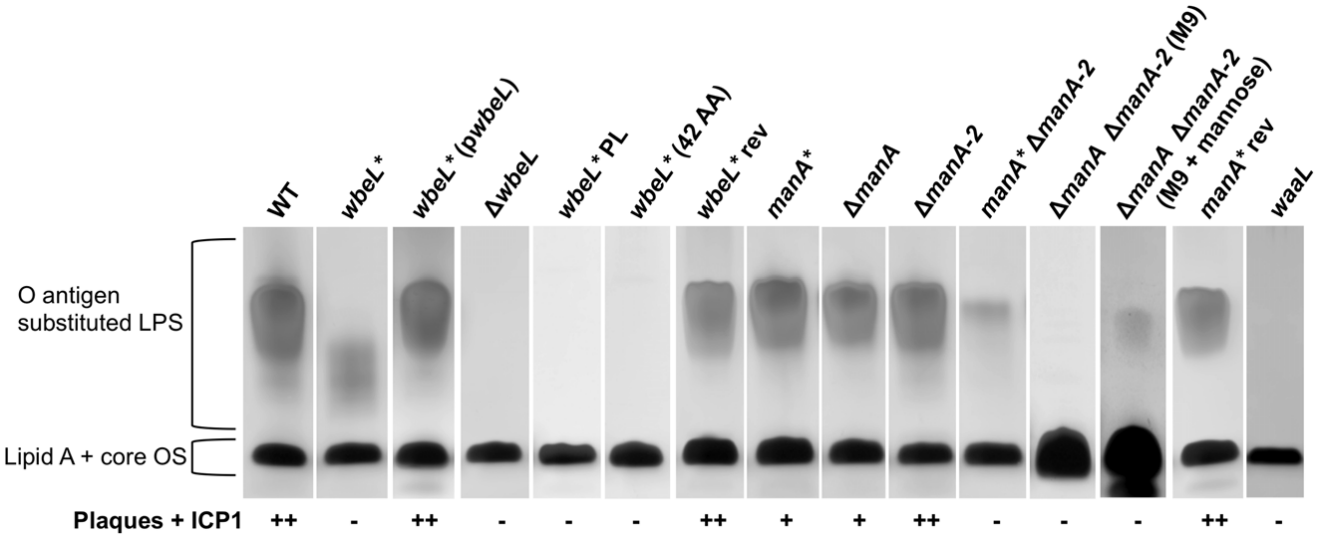
LPS profiles of wbeL* and manA* phase variants and related strains by SDS-PAGE and silver staining. Strains for LPS purification were grown overnight in LB (or M9-glucose +/− mannose as indicated) at 37°C with shaking. The bacterial strains are indicated above the lanes and the positions of the fully substituted LPS with attached O antigen and lipid A core oligosaccharide (OS) precursor are indicated. Approximately 5 µg of purified LPS was loaded into each lane, with the exception of the lanes containing LPS prepared from M9 cultures in which ∼30 µg of LPS was loaded. A strain lacking O1 antigen transferase activity, *waaL*, which has no O1 antigen, was used as a control for assessing the electrophoretic migration of the lipid A-core oligosaccharide. The ability of ICP1 to infect these strains as evidenced by plaque formation with bacteria grown to OD_600_∼0.5 is indicated as follows: (++) clear plaques, (+) turbid plaques, (−) no plaques.

Purified LPS from a wbeL* (A_7_) phase variant shows a distinct lower molecular weight pattern on a silver stained SDS-PAGE gel (Fig. 2), although the strain exhibits a normal slide agglutination phenotype with anti-Ogawa typing serum (Table 1). The wbeL* strain is completely resistant to infection with ICP1, and accordingly purified LPS from this strain shows no inhibition of ICP1 (Table 1). To test the possibility that the wbeL* A_7_ frame shift exerts a polar effect on downstream genes, which also contribute to tetronate biosynthesis (Fig. 1B), we performed complementation analysis and found that wild type LPS production and ICP1 sensitivity are restored when the wbeL* mutant is complemented with *wbeL in trans* (Fig. 2). This indicates that the observed phenotypes are a consequence of the loss of WbeL expression and not due to polar effects of the wbeL* mutation. To further confirm this, an in-frame deletion in *wbeL* was constructed and complementation analyses were performed. The Δ*wbeL* strain is devoid of O1 antigen (Fig. 2) and accordingly exhibited no agglutination with anti-Ogawa typing serum (Table 1), and these phenotypes can be complemented with *wbeL in trans* (data not shown). Since the phenotype of the in-frame deletion mutant is not consistent with the original wbeL* mutation, we hypothesized that the wbeL* allele maintains some O1 antigen biosynthetic function in the cell. Consistent with this, when the Δ*wbeL* strain is complemented with the wbeL* allele *in trans*, agglutination in the presence of anti-Ogawa typing serum is restored while the strain maintains complete resistance to ICP1, just like the original wbeL* strain (Table 1). The Δ*wbeL* mutant expressing wbeL* in trans also produces a small amount of lower molecular weight LPS like the original wbeL* strain (data not shown). To further address the biosynthetic function of the wbeL* allele, we explored two possible explanations for the phenotype of the wbeL* phase variant. First, that the presence of the truncated 42 amino acid peptide produced by the wbeL* allele is necessary and sufficient to allow *V. cholerae* O1 to elaborate the lower molecular weight LPS observed (Fig. 2). To test this hypothesis we constructed a deletion in the remainder of the coding sequence downstream of the premature stop codon in wbeL*. Purified LPS from a wbeL* strain expressing only the 42 amino acid peptide lacks O1 antigen substituted LPS (Fig. 2), which rules out this hypothesis. The second hypothesis that we tested to account for the biosynthetic role of the wbeL* allele is that it allows some functional WbeL protein to be made because the wbeL* allele is subject to nonstandard decoding (ribosomal frame shifting or transcriptional slippage at the A_7_ tract). This in turn would allow for a small amount of tetronate modification to occur, resulting in a lower molecular weight but still compositionally (and antigenically) normal O antigen (which is consistent with the observation that the wbeL* phase variant agglutinates in the presence of anti-Ogawa typing serum (Table 1). To test this hypothesis, we made silent point mutations within the A_7_ tract in wbeL* (A_7_ to TAAGAAA ) designed to prevent non-standard decoding as well as prevent further slipped-strand mispairing during replication and characterized the ability of this strain (referred to as wbeL* PL for phase-locked) to produce O1 antigen substituted LPS. The point mutations in wbeL* PL abolished the ability of this strain to make O antigen substituted LPS, which supports the conclusion that the wbeL* allele maintains biosynthetic function by allowing some functional WbeL protein to be produced through nonstandard decoding, and that this is dependent on the A_7_ tract. The mechanism responsible is not currently known, nor is it known if there are other sequence motifs within the wbeL* allele that facilitate the +1 frameshifting needed to restore the reading frame. It is important to emphasize that the observed lower molecular weight pattern of purified LPS from wbeL* is not due to reversion of A_7_ to A_8_ in the genome of a substantial subset of the population, because if that were the case we would observe a small amount of wild type length O1 antigen and not the unique species observed for the wbeL* strain. These results further suggest that tetronate acylation of the perosamine backbone is necessary for incorporation of the O1 antigen into the LPS molecule, and this may be required for recognition and subsequent transport of the undecaprenyl-linked O antigen polymer to the periplasm by the ABC transporter and/or for efficient ligation of the O antigen polymer to the lipid A-core by WaaL ligase [42]. Importantly, these data demonstrate that the lower molecular weight O1 antigen produced by the wbeL* mutant somehow endows *V. cholerae* with full resistance to ICP1.

**Table 1:** Summary of phenotypes of single and double mutants of *V. cholerae* O1 and their reverted or trans-complemented derivatives as analyzed by silver stained SDS-PAGE, phage sensitivity, and agglutination by anti-Ogawa typing serum.

Strain	O antigen substituted LPS^a^	ICP1 sensitivity^b^	Log_10_ inhibition of purified LPS^c^	Agglutination with serum
WT	++	++	2.3	+
*waaL*	None	−	n.d.	−
wbeL*	+	−	0	+
wbeL* p*wbeL*	++	++	n.d.	+
Δ*wbeL*	None	−	0	−
Δ*wbeL* p*wbeL*	++	++	n.d.	+
Δ*wbeL* pwbeL*	+	−	n.d.	+
wbeL* (42 AA)	None	−	n.d.	−
wbeL* PL	None	−	n.d.	−
wbeL* rev	++	++	n.d.	+
manA*	++	+	1.6	+
Δ*manA*	++	+	1.6	+
Δ*manA-2*	++	++	n.d.	+
manA* Δ*manA-2*	+	−	0	+
Δ*manA* Δ*manA-2* (in M9+glucose)	None	−	0	−
Δ*manA* Δ*manA-2* (in M9+glucose+mannose)	+	−	n.d.	+
*manA* rev	++	++	n.d.	++

a The pattern of purified LPS from strains grown overnight in LB (unless otherwise indicated) was visualized by SDS-PAGE and silver staining and the phenotype of each strain was categorized as follows: (++) wild type appearance of O antigen substituted LPS, (+) altered levels or appearance of O antigen substituted LPS, (None) no O antigen substituted LPS was observed.

b The ability of ICP1 to infect these strains as evidenced by plaque formation with bacteria grown to OD_600_∼0.5 is indicated as follows: (++) clear plaques, (+) turbid plaques, (−) no plaques.

c The ability of purified LPS to inhibit plaque formation by ICP1 was determined using 10 µg of purified LPS and is indicated as the average log_10_ inhibition of two independent assays. n.d. not done.

The second mutation identified in ICP1-resistant colonies from the center of plaques also mapped to a poly-A tract, and this localized to VC0269, which we designate *manA* for reasons explained below. *manA* has two poly-A (A_9_) tracts and, as illustrated in Fig. 1A, is located approximately 4 kbp downstream of the right junction gene (*rjg* = VC0264), which was thought to delineate the end of the O1 antigen biosynthetic locus in *V. cholerae*. ManA shows homology to type I PMIs that catalyze the reversible isomerization of F6P to M6P, which is the first step in perosamine biosynthesis (Fig. 1B). Like *wbeL*, *manA* is also specific to *V. cholerae* O1 strains: All 37 *V. cholerae* O1 strains available for bioinformatic analysis have *manA* and it is highly conserved between these strains with the exception of two strains which have the manA* (A_8_) allele resulting from a single nucleotide deletion in the first poly-A tract (these latter strains are designated 2740–80 and HC-61A1). The full length ManA protein is 399 amino acids, and a single nucleotide deletion within the first poly-A tract is predicted to result in a truncated peptide 81 amino acids long, while a single nucleotide deletion within the downstream poly-A tract (designated manA*) produces a truncated peptide 207 amino acids long (Fig. S3). LPS purified from manA* after overnight growth looks identical to the parental strain (Fig. 2). However, purified LPS from the manA* strain does not bind and inhibit ICP1 as efficiently as a equal amount of LPS purified from the parental strain (Table 1), which is consistent with this mutant being partially phage resistant (Fig. 2 and Table 1). These results leave open the possibility that while manA* produces wild type length O1 antigen, the overall abundance of O1 antigen substituted LPS is less than wild type, and this translates into fewer available receptors for ICP1. Phage infection is a complex process, which can require sequential receptor binding steps. For example T4 infection is initiated by the reversible attachment of at least three of the six long tail fibers to the outer core of the LPS, but does not result in DNA injection unless the six short tail fibers successfully engage their receptors on the inner core of the LPS [43]–[45]. Throughout the course of this study isolates were obtained with a frameshift in either poly-A tract in *manA* and these isolates were phenotypically indistinguishable from one another with regard to phage sensitivity and agglutination with anti-Ogawa typing serum (data not shown). Similarly, a strain harboring an in-frame deletion of *manA* was phenotypically indistinguishable from the *manA* phase variants (Fig. 2 and Table 1), indicating that both frame-shift mutations function as *manA* nulls.

### *manA* and *manA-2*, but not *manC*, are important for perosamine biosynthesis

*manA* does not appear to be required for producing full length O antigen as indicated by SDS-PAGE and silver staining of LPS (Fig. 2), and yet the manA* strain is partially resistant to ICP1, which specifically requires the O1 antigen for infection. These observations led us to speculate that there is likely another gene that can contribute to the conversion of the F6P to M6P in *V. cholerae* O1. We noted the presence of another annotated type I PMI in the *V. cholerae* O1 genome, VC1827, hereafter designated *manA-2*. *manA-2* is located immediately downstream of a mannose permease encoded by VC1826 [46], but is not linked to the O1 antigen biosynthetic cluster. *manA* and *manA-2* are 65% identical at the nucleotide level over 70% of their sequence, and at the protein level, they are 59% identical over 97% of their sequence. *manA-2* has a higher GC content than *manA* (46.1% compared to 42.1%, respectively) and its GC content is much closer to the overall GC content of the entire *V. cholerae* N16961 genome (∼47% [47]) suggesting *manA* may have been recently horizontally acquired. Unlike *manA*, *manA-2* is found in non-O1 *V. cholerae*. In other Gram-negatives, including *E. coli* and *S*. Typhimurium, the *manA* gene generally maps as an independent gene not associated with the LPS gene cluster due to its role in mannose metabolism [48], [49], although in these organisms the PMI activity of the unlinked *manA* is required for O antigen synthesis [50], [51]. In organisms that do not metabolize mannose, the *manA* gene is generally absent [49]. Some bacteria have a bifunctional type II PMI-GMP (typically referred to as ManC) encoded in the LPS biosynthetic cluster. Monofunctional and bifunctional forms of ManC are not clearly distinguishable on the basis of size or even sequence similarity [49]. Indeed ManC (VC0241) in *V. cholerae* O1 has the bioinformatic designation of a type II PMI, however our results below suggest this enzyme lacks PMI activity.

The presence of two putative type I PMIs in *V. cholerae* O1 led us to investigate the phenotypes of single and double mutants with regards to O antigen biosynthesis. A mutant lacking *manA-2* was indistinguishable from wild type with regard to phage sensitivity and LPS pattern on a gel (Fig. 2 and Table 1), indicating that *manA-2* is not important for O antigen biosynthesis under the conditions tested. However, in the absence of *manA*, *manA-2* becomes important since the manA* Δ*manA-2* double mutant is completely phage resistant and produces very little fully length LPS (Fig. 2). Another potential source of M6P for perosamine biosynthesis is through the conversion of exogenously acquired mannose to M6P by a hexokinase, which led us to determine if trace mannose present in the growth media (Luria-Bertani [LB] broth) contributed to the small amount of O1 antigen still visible in the manA* Δ*manA-2* double mutant. Indeed, this small amount of O1 antigen substituted LPS is absent when the double mutant is grown in M9-glucose, but is present when the double mutant is grown in M9-glucose plus mannose (Fig. 2), demonstrating that exogenous mannose is responsible for the small amount of O1 antigen substituted LPS in the double mutant. Similar results have been observed in *E. coli* and *S.* Typhimurium *manA* mutants, which are unable to synthesize O antigen without the inclusion of mannose in the growth media [50], [51]. Also consistent with these results, we observed that our double manA* Δ*manA-2* mutant is unable to grow in M9-mannose owing to the critical nature of type I PMIs in the conversion of M6P to F6P as a substrate for glycolysis. These results indicate that *manC* in *V. cholerae* O1, which was hypothesized to catalyze the first reaction in biosynthesis of perosamine (Fig. 1B) [12], is not active as a bifunctional PMI-GMP, and is likely only important for the later steps in perosamine biosynthesis for converting M1P to GDP-mannose. As type II PMIs possess two catalytically distinct domains for each PMI and GMP activity [20], attempts were made to construct mutant *manC* alleles that should be defective for PMI activity alone (data not shown) but all constructs resulted in complete depletion of the O1 antigen, suggesting that these mutations had an inadvertent negative impact on the GMP activity of the protein. The reason behind *V. cholerae* O1 possessing two type I PMIs is thus not clear, although it suggests that they each have their primary roles: *manA* in O antigen biosynthesis and *manA-2* in mannose metabolism, though it is unclear what factors define those functional roles. A search for other bacteria harboring multiple annotated type I PMIs reveals a limited number of organisms including strains of *Vibrio vulnificus* (Accession No. NC_005140), *Vibrio parahaemolyticus* (NC_004605), and *Yersinia enterocolitica* (NC_015224); however, to our knowledge, the functional roles that these enzymes have in these other organisms are not known.

As mentioned previously, the O1 antigen biosynthetic cluster was originally identified through the heterologous expression of the *V. cholerae* O1 antigen in *E. coli* [10], and all O1 antigen biosynthetic genes studied thus far have been between the *gmh*D and *rjg* flanking genes. *manA* was likely not identified as part of this biosynthetic pathway because its function was complemented by the *E. coli manA* gene, permitting expression of the O1 antigen in this host. Additional genes required for O1 antigen biosynthesis were subsequently identified following the initial report by Manning *et al.* [11], and were similarly likely missed because the phenotype was masked in *E. coli*. Blokesch and Schoolnik [52] provided some additional support that further extends the O1 *wbe* region downstream of *rjg*. They observed that when serogroup conversion of *V. cholerae* O1 to O139 occurred through uptake of O139 donor DNA during natural transformation, the crossovers were often localized within or downstream of VC0271 at the right junction, and the location of the left junction was within or upstream of *gmhD*. These results coupled with our observation that *manA* participates in O1 antigen biosynthesis suggests that the *wbe* region extends approximately 8 kbp downstream of *rjg* (Fig. 1B). There are six genes currently annotated in addition to *manA* in this region (Fig. 1A), however it remains to be seen if these other genes do in fact participate in O1 antigen biosynthesis.

### *V. cholerae* O1 *wbeL* and *manA* phase variants are defective for colonization of the small intestine

**Figure 3:**
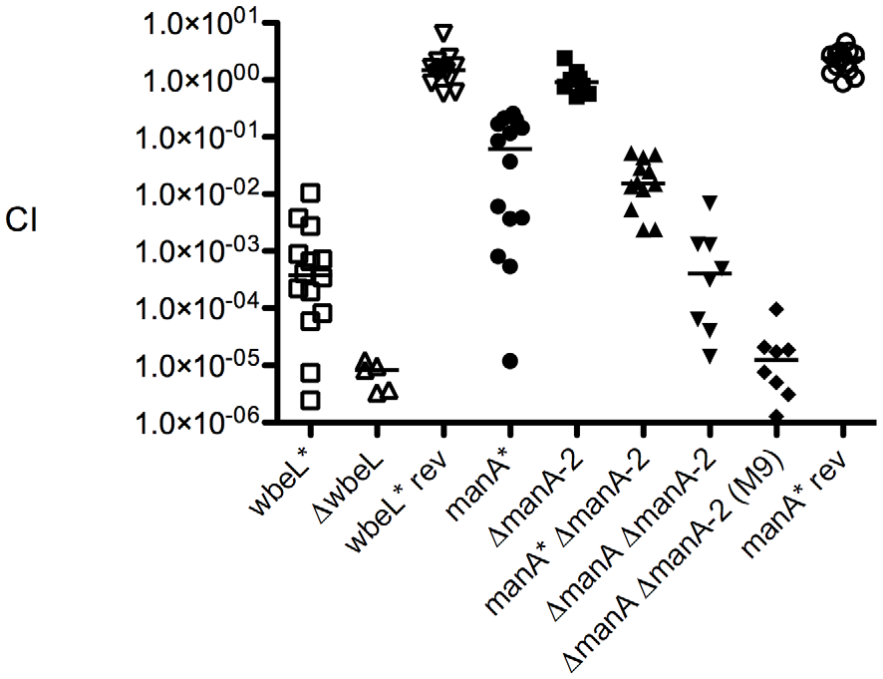
*V. cholerae* O1 phase variants are attenuated *in vivo*. Competition indices (CI) were determined between the strains indicated and the parental strain carrying a *lacZ* deletion in infant mice 24 hours post infection. Each symbol represents the CI for an individual mouse and horizontal lines indicate the median CI for each strain.

The ability of wbeL* and manA* phase variants to colonize the small intestine was assessed in competition assays in the infant mouse model. The wbeL* strain is attenuated over 1000-fold (Fig. 3). We did not anticipate such a high level of attenuation given that this strain still elaborates LPS (although it is a lower molecular weight form, Fig. 2). Interestingly, the altered LPS produced by the wbeL* strain does provide some advantage over not having any O1 antigen, as is apparent by the significantly lower competitive index (CI) for the Δ*wbeL* strain (p<0.05, Mann-Whitney U test). To rule out secondary mutations, we chose to use revertant strains *in vivo* to avoid potential complications concerning plasmid loss and non-wild type gene expression levels during infection. The colonization defect observed for the wbeL* strain is absent when the poly-A (A_7_) tract is reverted to wild type length (A_8_) (Fig. 3), demonstrating that the virulence defect is due to the wbeL* allele. The manA* phase variant, which produces an apparently full length LPS but which is partially resistant to ICP1, is over ten-fold attenuated for colonization, and this defect is absent when the A_8_ tract is reverted to wild type length (A_9_) (Fig. 3).

We anticipated that a manA* Δ*manA-2* double mutant would be significantly more attenuated owing to a major reduction in O1 antigen produced by the strain (Fig. 2). However, although we did observe a further decrease in the CI for manA* Δ*manA-2* compared to manA*, it was not significant (p = 0.42) (Fig. 3). To try and explain this, we hypothesized that the selective pressure exerted on the manA* Δ*manA-2* double mutant in the small intestine is sufficient to select for spontaneous revertants which would potentiate the observed moderate drop in CI. To test this, we patched manA* Δ*manA-2* isolates recovered from mouse intestines onto M9-mannose agar plates. Consistent with our hypothesis, roughly half of isolates recovered from mice after 24 h of infection had regained the ability to grow on mannose, and subsequent sequencing revealed that these strains had reverted to the wild type, in-frame poly-A tract in *manA*. Conversely, we were unable to detect spontaneous revertants of the wbeL* or manA* strains among the isolates recovered from mouse intestines infected with those strains, suggesting that the A_7_ wbeL* allele is less prone to reversion by slipped-strand mispairing, and that the A_8_ allele in manA* is not under enough selective pressure to revert as long as ManA-2 is functional. Our inability to detect wbeL* revertants is likely due to the experimental limitation that there are so few wbeL* isolates recovered from infected mice that we were only able to test ∼100 CFU.

The *manA* revertants recovered from intestines of mice infected with the manA* Δ*manA-2* strain were effectively equivalent to Δ*manA-2*, which we previously observed has no effect on LPS biosynthesis or virulence (Fig. 2 and Fig. 3). Confirming this, we also competed the double mutant with in-frame deletions in both *manA* and *manA-2* and observed the anticipated significant increase in attenuation (>2000-fold) compared to a manA* phase variant alone (p<0.05 Mann-Whitney U test) (Fig. 3). Furthermore, the double Δ*manA* Δ*manA-2* mutant is significantly more attenuated when grown in M9-glucose prior to infection than when it is grown in LB which contains trace mannose (p<0.05 Mann-Whitney U test). Consistent with this, a comparable CI was observed for the two strains that are devoid of all O1 antigen prior to infection (Δ*manA ΔmanA-2* grown without exogenous mannose, and Δ*wbeL*) (Fig. 3). *In vitro* control competitions revealed that the observed defects were specific to the *in vivo* environment, with the exception of the Δ*wbeL* mutant, which has a ∼10-fold defect *in vitro* compared to the wild type (data not shown). The nature of this defect is not known, but it could be due to the build-up of O antigen and/or LPS intermediate products in this strain which, unlike the wbeL* phase variant, is unable to elaborate any O1 antigen on the surface. Consistent with this, mutants harboring transposon insertions in *wbeL*, which also showed a decreased colonization phenotype in infant mice, also exhibited a decreased CI in *in vitro* control competitions [53].

### *V. cholerae* O1 phase variants are sensitive to the antimicrobial peptide polymyxin B

Human intestinal epithelial cells produce antimicrobial peptides that are critical components of the host innate defense mechanism [54]. Antimicrobial peptides are inherently structured to target the membrane of bacteria because they are highly basic and have a substantial portion of hydrophobic residues [55], [56]. The net positive charge of these peptides facilitates their electrostatic interaction with negatively charged phospholipid groups or the lipid A anchor of LPS on the Gram-positive or Gram-negative bacterial membranes, respectively, allowing them to induce lysis and bacterial cell death. In order for an antimicrobial peptide to gain access to the Gram-negative outer membrane it must first traverse the barrier presented by the sugar chains of the O antigen layer. Perturbations to the LPS have been shown previously to alter resistance of *V. cholerae* to antimicrobial peptides; specifically, Matson *et al.* [57] and Hankins *et al.* [58] have investigated the structural importance of lipid A with regards to peptide resistance, and Nesper *et al.* [59] suggested that mutations affecting the LPS core oligosaccharide have a more dramatic affect on antimicrobial peptide resistance than mutations affecting O1 antigen biosynthesis (although only a rough strain was tested in those experiments).

**Figure 4:**
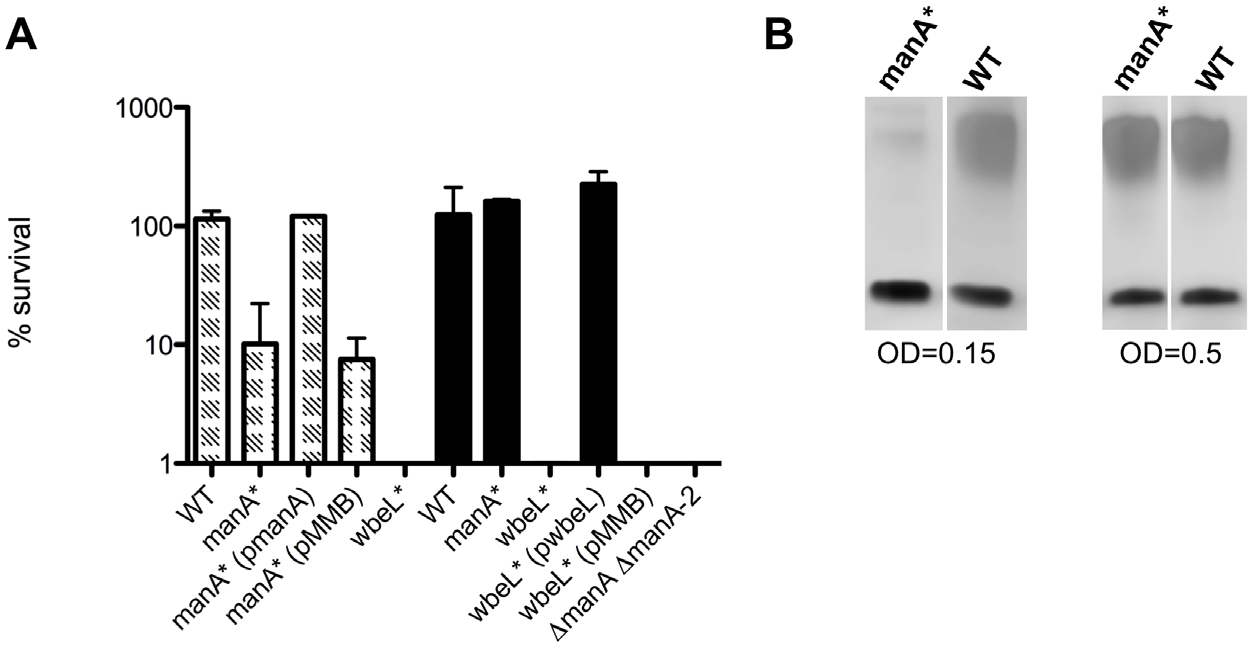
*V. cholerae* O1 phase variants are more sensitive to the antimicrobial peptide polymyxin B. A) Strains were grown to OD_600_ = 0.15 (hatched columns) or OD_600_ = 0.5 (solid black columns) and exposed to 50 µg/ml polymyxin B for 2 hours. After treatment, serial dilutions of each culture were plated for enumeration to determine the percent survival compared to a mock-treated control. Data represents the means and standard deviations of two independent experiments performed in technical duplicate. B) manA* phase variant exhibits O1 antigen production dependent on growth phase. Approximately 2 µg of purified LPS was loaded into each lane.

We investigated the susceptibility of wbeL* and manA* phase variants and their trans-complemented derivatives to the antimicrobial peptide polymyxin B by determining their survival in killing assays. We observed that the wbeL* mutant exhibited a very low level of survival (Fig. 4A). This phenotype could be complemented and survival levels could be restored to wild type when this mutant was expressing *wbeL in trans*, again supporting the previous data suggesting there is no polar effect of wbeL* on genes downstream. With regards to the manA* phase variant, we observed polymyxin sensitivity, but in a growth phase-dependent manner. There was intermediate sensitivity of this strain to polymyxin B when the inoculum used for the killing assay was grown up to early exponential phase (OD_600_ = 0.15) (Fig. 4A). In contrast, when manA* was grown up to mid-exponential phase prior to treatment with the peptide, wild type levels of survival were observed (Fig. 4A). The phenotype observed at early exponential phase with the manA* phase variant could be fully complemented by expressing *manA in trans* (Fig. 4A), again indicating the observed phenotypes are due to the loss of ManA. We had also observed that phage sensitivity of the manA* phase variant was growth phase-dependent, and these results parallel the observations in the antimicrobial peptide assay, that is at OD_600_ = 0.15, manA* is completely resistant to ICP1 and at OD_600_≥0.2, turbid plaques result from ICP1 infection (data not shown). To address this puzzling observation, we purified LPS from manA* and wild type at early and mid-exponential growth phase (OD_600_ = 0.15 and OD_600_ = 0.5, respectively). In contrast to the seemingly wild type appearance of purified LPS from manA* after overnight growth (Fig. 2) and at mid-exponential phase, the LPS pattern of manA* isolated at early exponential growth phase showed very little O1 antigen substituted LPS (Fig. 4B). Since analysis of the double manA* Δ*manA-2* mutant indicated that *manA-2* is important for O1 antigen synthesis only in the absence of *manA*, we interpret these results to suggest the compensatory activity of ManA-2 is incomplete during early exponential phase growth in LB broth. The reason for this is not known, but may relate to differences in expression, activity or localization of ManA-2. In any event, a complete functional redundancy between ManA and ManA-2 would have been at odds with the observation that all *V. cholerae* O1 strains have a phase variable *manA* gene: Specifically, the evolution of a contingency locus would be futile if the encoded protein exhibited complete functional redundancy with a non-phase variable gene.

In general, the results of the polymyxin B killing assays (Fig. 4A) parallel the observed *in vivo* colonization defects (Fig. 3). Strains that produce less O1 antigen substituted LPS (such as wbeL* and Δ*manA* Δ*manA-2*) are highly susceptible to polymyxin B and are more severely attenuated *in vivo* than manA*, which accordingly is less susceptible to polymyxin B. These data suggest that the phase variants are defective for *in vivo* colonization because they are more susceptible the antimicrobial peptides present in the intestinal tract.

### Phase variation of *wbeL* occurs as the dominant mechanism of O1 antigen variation in the face of predation by phage in a simulated natural environment

*V. cholerae* O1 persists in the environment as a member of the aquatic ecosystem where it is thought to associate with and use the chitinous exoskeletons of zooplankton as a nutrient source [60]. The levels of *V. cholerae* O1 phages in the environment, including potentially ICP1, have been shown to inversely correlate with disease burden suggesting that phage predation in the natural environment may contribute to the collapse of a given cholera epidemic [41]. We investigated the potential for phage resistance to develop in a simulated natural environment comprised of chitin and pond water. A thousand CFU from independent cultures of wild type *V. cholerae* O1 were inoculated into pond water with chitin, and ICP1 was added at an MOI of 0.01. After 24 hours at 30°C, we observed that in all 11 pond microcosms to which ICP1 was added, the phage titer increased at least one million-fold (data not shown). Bacterial levels in the uninfected control had increased 10,000-fold, while the bacterial levels in the infected microcosms varied substantially from below the detection limit to levels nearly comparable to that observed in the uninfected control (Table 2). All isolates recovered from environments to which phage was added were resistant to ICP1 infection. Furthermore, the majority of isolates (63 out of 80 isolates, 79%) from independent microcosms that had become resistant to ICP1 had the wbeL* frameshift allele (Table 2). We only observed heterogeneity in the resistance mechanism for isolates from one microcosm (Table 2, number 10), suggesting that in most cases phage predation resulted in the clonal expansion of a single resistant mutant. We did not observe any mutations in the poly-A tracts of *manA*, likely owing to the incomplete ICP1 resistance afforded to *V. cholerae* with those alleles. Control experiments with manA* and wbeL* variants as input strains in the absence of phage confirmed that neither mutant exhibit a decreased ability to grow in the simulated pond microcosm (data not shown). Isolates from all microcosms to which phage were added were also replica plated onto LB agar containing 350 µg/mL polymyxin B (a concentration that permits growth of the wild type parent but not wbeL* or manA*), and all isolates were unable to grow. This indicates that the mutations that occurred less frequently and that did not map to *wbeL* also likely affected LPS biosynthesis. These results show that in a simulated natural environment phage predation can occur with consequent selection for bacteria with altered O1 antigen, and that the dominant mechanism by which mutational escape is achieved is through mutations in the poly-A tract in *wbeL*.

**Table 2:** Phage predation in a pond microcosm leads to the selection of wbeL* phage resistant mutants.

Microcosm^a^	Phage added^b^	Total CFU 24 h p.i.^c^	% WT^d^	ICP1 resistant isolates		
				% wbeL*	% manA*	% Other
1	−	2×10^7^	100	0	0	0
2	+	4×10^6^	0	100	0	0
3	+	4×10^5^	0	100	0	0
4	+	1×10^6^	0	100	0	0
5	+	4×10^3^	0	0	0	100
6	+	4×10^5^	0	100	0	0
7	+	6×10^5^	0	100	0	0
8	+	<d.l.	−	−	−	−
9	+	1×10^7^	0	0	0	100
10	+	6×10^5^	0	87.5	0	12.5
11	+	6×10^5^	0	100	0	0
12	+	4×10^5^	0	100	0	0

a Independent cultures of wild type *V. cholerae* O1 were used to inoculate 10^3^ CFU per microcosm containing pond water and chitin as a sole nutrient source.

b ICP1 was simultaneously added at an MOI = 0.01 where indicated.

c <d.l. is below the limit of detection for this assay (1 CFU).

d Eight isolates from each microcosm were tested for resistance to ICP1. If no alteration to sensitivity was observed, the isolates were categorized as WT. If resistance was observed than the poly-A tracts in *wbeL* and *manA* were sequenced. If resistance was observed and a frameshift in neither *wbeL* nor *manA* was observed, than the isolates were categorized as other.

We also investigated the diversity of phage resistant mutants that appeared in the center of plaques on LB plates by similarly sequencing the poly-A tracts in *wbeL* and *manA* in 83 phage resistant isolates. In contrast to the experiment designed to mimic the natural aquatic environment, during selection in LB soft agar overlays in which diffusion of phage and bacteria are relatively limited, the mode of phage resistance is much more varied although a substantial portion can be attributed to phase variation in *wbeL* and *manA* (∼20%); we found that six out of 83 isolates were wbeL* phase variants and 11 out of 83 were manA* phase variants (six of the 11 had a deletion that mapped to the first poly-A tract, and the other five to the second poly-A tract). The reason for this difference in frequency of wbeL* and manA* occurrence compared to the pond microcosm is not known. However, as was mentioned previously (and will be addressed below) the manA* mutation was observed in sequenced *V. cholerae* O1 isolates; therefore it is apparent there are relevant circumstances in which manA* phase variants are selected for.

### O1 antigen heterogeneity exists between clinical isolates of *V. cholerae* O1

We wanted to determine if O antigen heterogeneity exists in the population of *V. cholerae* excreted along with ICP1 phage from patients during natural infection. To do this we obtained three ICP1-positive stool samples collected from three patients admitted to the International Centre for Diarrhoeal Disease Research, Bangladesh (ICDDR,B) during a cholera epidemic in 2001. Several representative isolates from all three stool samples were analyzed and were found to be *V. cholerae* O1 Inaba and were sensitive to ICP1. We tested the ability of these stool isolates to become phage resistant by collecting phage resistant mutants from the centers of plaques resulting from ICP1 infection and readily isolated manA* and wbeL* phase variants (data not shown), showing that these clinical isolates can phase vary at these contingency loci. Next we screened many thousands of colonies from each of these archived stool samples for the presence of manA* and wbeL* phase variants, which we hypothesized might have arisen as a result of ICP1 phage pressure during the patient infection. Since we showed above that manA* and wbeL* phase variants are attenuated in the infant mouse model, we expected that their frequency in the human stool samples would be low or perhaps even undetectable due to decreased fitness during infection of the human small intestine. As we had done previously, we took advantage of the observation that both phase variants exhibit increased sensitivity to polymyxin B compared to the parental strain. We determined that both the wbeL* and manA* phase variants obtained *in vitro* from these clinical isolate strain backgrounds failed to grow on LB agar plates containing 800 µg/ml polymyxin B after replica plating, while the parental strain maintained its ability to grow. We screened approximately 5,000 colonies from each stool sample by replica plating and did not observe any isolates with increased polymyxin B sensitivity.

In the above analysis we were limited by the number of available archived stool samples, since routine practice is to purify a single colony from a stool sample and store that for further examination. While such single colony isolate collections cannot be used to answer the question of whether O antigen heterogeneity exists within the population of *V. cholerae* excreted from a single patient, it does allow us to determine if O antigen heterogeneity exists between isolates recovered from different patients. We evaluated phage sensitivity of approximately 50 isolates recovered from cholera patients at the ICDDR,B between 2001 and 2005. Three of these isolates displayed resistance to ICP1 and were characterized further. One of the clinical isolates has a mutation in the second poly-A tract in *manA*. The other two resistant isolates did not have mutations that mapped to the poly-A tracts in either *manA* or *wbeL*, and examination of purified LPS from these strains showed that they produce very little full length O-antigen substituted LPS (data not shown), however the nature of the observed defects is not known.

From these results it appears that, despite the hypothesized ICP1 phage pressure during infection and despite pressure from the host immune system to reduce or alter the O1 antigen (O1 antigen is the dominant antigen [61], [62]), the intra-patient and inter-patient O1 antigen variability is quite low. In agreement with this, previous studies have detected LPS mutants in mice following coinfection of phage and *V. cholerae* O1, but at very low frequencies. Zahid *et al.*, [63] estimated that the frequency of such mutants was on the order of 10^−8^, and in accordance with our results, failed to detect phage-resistance heterogeneity among *V. cholerae* O1 directly from human stools. Additionally, despite relatively high levels of O1-specific phage in the stool samples, *V. cholerae* O1 isolated from the same stool samples remain completely susceptible to phage lysis [63]–[65].

Previous studies of *V. cholerae* O antigen negative strains or strains with altered LPS structures were also found to be defective in colonization of infant mice [53], [59], [63], [66]–[69]. These results support the assertion that O1 antigen-deficient *V. cholerae* would be selected against during human infection due to the high fitness cost associated with mutational escape. When put into the context of our findings, it is fitting that mutational escape is frequently conferred through phase variability, as a key feature defining this mode of variability is its reversible nature [23], [32], [70]. Inherently each cell will retain its ability to switch between expression states (the switching rates of phase variable genes are typically between 10^−2^ to 10^−5^ [32]), and therefore the phenotype of a clonal population of bacteria capable of phase variation will vary as a function of selection. Predation of *V. cholerae* O1 in the environment by O1 antigen-dependent lytic phage may rapidly select for the subpopulation with altered O1 antigen mediated by manA* and wbeL* frame-shifted contingency loci, and even though those subpopulations are less suited for life in the intestinal tract, positive selection (for example when this mixed population is ingested by a human) results in enrichment of the subpopulation with full O1 antigen expression. We were able to experimentally confirm the reversibility of frameshift mutations occurring at the poly-A tract in *manA*, however, we were unable to do so for *wbeL* likely due to experimental limitations (discussed above). It is possible that the observed mutational escape mediated by the poly-A tract in *wbeL* is a function of hypermutation and not phase variation (if it is not reversible). However, we have clearly demonstrated the utility of these loci in mediating alterations in the expression of the key *V. cholerae* antigen and phage receptor.

### Concluding comments

Pathogenic *V. cholerae* O1 has evolved to live in very diverse environments including fresh water, salt water and the human small intestine. The O1 polysaccharide antigen is the dominant cholera antigen and can induce protective immune responses in humans and animals [61], [71]–[73], and thus is a critical immunogen guiding cholera vaccine development. The extent to which *V. cholerae* can vary expression of the O1 antigen is not currently appreciated. We demonstrate for the first time that the O1 antigen is subject to phase variation and show that this is mediated by three homonucleotide tracts in two genes (*wbeL* and *manA*), which are critical for O1 antigen biosynthesis. The ubiquitous presence of these phase variable homonucleotide tracts in all *V. cholerae* O1 strains points to the significant role they play in modulating expression of this surface exposed antigen. Moreover, by identifying *manA* as critical for O1 antigen biosynthesis, we have extended the genome boundaries previously believed to contain all the necessary genes for O1 antigen biosynthesis in *V. cholerae*. Phase variation mediated by homonucleotide tracts has not been previously well-documented in *V. cholerae*. To our knowledge, the only prior report of phase variation in *V. cholerae* was that by Carroll *et al.*, [74] in which expression of the membrane bound virulence regulator, TcpH, was observed to be subject to phase variation mediated by a poly-G (G_9_) tract. However, with the growing list of currently available *V. cholerae* O1 genome sequences, it is clear that this tract is not well-conserved (only three of the available 37 sequenced strains have this tract [data not shown]), and thus this likely does not represent a wide-spread mechanism employed by *V. cholerae* O1 to alter virulence expression. Examination of the currently available *V. cholerae* O1 genome sequences may facilitate further exploration of phase variation in this organism; it is interesting to note that there are only twelve homonucleotide tracts of nine or greater nucleotides in length located within coding regions in the *V. cholerae* O1 N16961 genome, and several of these are located within known virulence factors (data not shown), however the significance this remains to be examined.

The biological role of phase variation in mucosal pathogens is frequently anticipated to facilitate immune evasion in the host [75]. However, in the case of the facultative pathogen *V. cholerae*, our data point to the primary role for O1 antigen phase variation as a strategy for dealing with the strong opposing selective pressures of phage predation in the environment and the strict requirement of O1 antigen for colonization of the intestinal tract. Phase variation of these genes thus allows for a subset of the population of *V. cholerae* being disseminated from a patient or being ingested in contaminated water, to be resistant to O1-dependent phages or to be virulent, respectively, thus contributing to the overall fitness of this pathogen. We hypothesize that the host immune response represents yet a second strong selective pressure against the O1 antigen, though the effects of this on circulating strains of *V. cholerae* within immune populations has not been studied.

The ubiquitous presence and overall success of ICP1-related phages is likely, at least in part, due to their use of a critical virulence factor as a receptor [40]. Our observation that mutational escape facilitated by *wbeL* and *manA* predominates *ex vivo* strongly suggests that ICP1 is particularly adept at predation of *V. cholerae* O1 within the human host where the requirement for colonization and virulence necessitates the maintenance of the O1 antigen. This may suggest a mechanism whereby this phage and the human host act synergistically to limit *V. cholerae* during infection, and perhaps how phage contribute to the overall decline of a given cholera epidemic as has been hypothesized [41], [64]. It remains to be seen if there are additional mechanisms employed by *V. cholerae* O1 to evade phage predation, specifically within the human intestinal tract, and how this arms race between ICP1 and its bacterial host shapes the evolution of the circulating *V. cholerae* O1 strains within the endemic region of Bangladesh.

## Materials and Methods

### Growth conditions

Strains were grown on Luria-Bertani (LB) agar or in LB broth at 37°C with 100 µg/ml streptomycin (Sm). When indicated M9 minimal media (supplemented with trace metals, vitamins (Gibco MEM Vitamins, Invitrogen), 0.1% casamino acids) with 0.4% glucose and/or 0.4% mannose was used. Strains containing the pMMB67EH vector were grown in the presence of 100 µg/ml Sm and 50 µg/ml ampicillin (Amp). Expression from the P_tac_ promoter was induced by the addition of 1 mM isopropyl-β-d-thiogalactopyranoside (IPTG). Phage susceptibility was determined by the soft agar overlay method as described previously [40] and/or by measuring growth of a bacterial isolate in the presence of ICP1 (to an approximate MOI = 1) in LB plus Sm broth culture using a Bio-Tek microplate reader.

### Isolation of spontaneous *manA* and *wbeL* phase variants and whole genome resequencing

A wild type *V. cholerae* O1 strain (E7946) was used in standard plaque assays with phage ICP1 as previously described [40]. Following overnight incubation, colonies were routinely observed in the center of plaques indicating the presence of phage resistant isolates. Four independent colonies were chosen for further analysis including phage resistance assays and whole genome sequencing using an Illumina genome analyzer II (Tufts University Core facility) as previously described [40]. Assembled genomes were aligned to the *V. cholerae* O1 N16961 [47] and E7946 (unpublished data) reference genomes. Two of the independently isolated phage resistant strains had a single nucleotide deletion in the poly-A tract of *wbeL* (designated wbeL*), while one phage resistant derivative had a single nucleotide deletion in the second poly-A tract of *manA* (designated manA*). The other derivative not chosen for further study had a nonsynonymous substitution in *manB*.

### Bacterial strain construction

PCRs for sequencing and cloning were carried out using EasyA polymerase (Agilent). Primer sequences are available upon request. In-frame unmarked deletions were constructed using splicing by overlap extension (SOE) PCR [76] and introduced using pCVD442-lac [77]. Deletion alleles constructed in this study are missing the entire open reading frame, except for the start and stop codons (with the exception of the *wbeL* deletion allele which also preserved a single codon immediately upstream of the stop codon). Expression plasmids were constructed by cloning the desired open reading frame(s) (including the predicted ribosome binding site) into the multiple cloning site of pMMB67EH. Expression vectors were transferred into *V. cholerae* by conjugation with *E. coli* SM10λpir and selection of Sm^R^ Amp^R^ colonies. Strains utilized in this study are shown in Table 3.

**Table 3:** Strains used in this study.

*V. cholerae* strains	Relevant genotype/description	Reference or Source
WT (E7946)	Spontaneous SmR derivative of E7946, El Tor Ogawa	[79]
wbeL*	Spontaneous phage resistant isolate, single nucleotide deletion in poly-A tract (A_8_→A_7_) in *wbeL* (VC0249)	This study
Δ*wbeL*	In-frame deletion of *wbeL*	This study
wbeL* (p*wbeL*)	wbeL* (pMMB67EH::*wbeL*)	This study
Δ*wbeL* (p*wbeL*)	Δ*wbeL* (pMMB67EH::*wbeL*)	This study
Δ*wbeL* (pwbeL*)	Δ*wbeL* (pMMB67EH::wbeL*)	This study
wbeL* (42 AA)	wbeL* with a deletion in the remainder of the coding sequence downstream of the premature stop codon	This study
wbeL* PL	wbeL* with nucleotide substitutions within the A_7_ tract (A_7_ to TAAGAAA )	This study
wbeL* rev	Reversion of wbeL* to wild type *wbeL*	This study
manA*	Spontaneous phage resistant isolate, single nucleotide deletion in second poly-A tract (A_9_→A_8)_) in *manA* (VC0269)	This study
Δ*manA*	In-frame deletion of *manA* (VC0269)	This study
Δ*manA-2*	In-frame deletion of *manA*-2 (VC1827)	This study
manA* Δ*manA-2*	In-frame deletion of *manA*-2 (VC1827) in manA* background	This study
Δ*manA* Δ*manA-2*	In-frame deletion of *manA*-2 (VC1827) in Δ*manA* background	This study
manA* rev	Reversion of manA* to wild type *manA*	This study
manA* (p*manA*)	manA* (pMMB67EH::*manA*)	This study
Δ*lacZ*	In-frame deletion of *lacZ*	[80]
*waaL*	*waaL*::pGp, *V. cholerae O1* SV194 background, O1 antigen minus	[42]
Bacteriophage		
ICP1	O1-specific phage of *V. cholerae* O1	[40].

### LPS analysis

Slide agglutination tests were performed using *V. cholerae* O1 Ogawa polyclonal rabbit antiserum (Difco). LPS was extracted from overnight cultures unless otherwise indicated, as described previously [72]. Briefly, cultures were centrifuged and washed twice in TM buffer (50 mM Tris [pH 7.5], 10 mM MgCl_2_) supplemented with 1 mM DL-Dithiothreitol before being lysed by bead-beating (BioSpec Products, Inc.) with 0.1 mm zirconia beads for a total of three minutes with intermittent incubations on ice. Whole cell lysates were treated with proteinase-K (Sigma) at 37°C for 24–48 h as required. Phenol extraction was performed using phase-lock gel light tubes (Eppendorf). Extracts were centrifuged at 75,000× g for 60 min, the pellet was washed with TM buffer and centrifuged as before. Purified LPS was separated on a 4–12% NuPage Bis-Tris gel (Invitrogen) and visualized by silver-staining (SilverQuest, Invitrogen). The concentration of *V. cholerae* LPS was determined by comparison to a standard curve of *E. coli* O26:B6 LPS (Sigma) using a Fujifilm FLA-900 scanner as previously described [72]. The ability of purified LPS to neutralize plaque formation was determined as previously described [40].

### Infant mouse colonization assays

*In vivo* competition experiments were done using 4–5 day old CD-1 mice. The dams and their litters were housed with food and water *ad libitum* and monitored in accordance with the rules of the Department of Laboratory Animal Medicine at Tufts Medical Center. The inoculum was prepared as a 1∶1 mixture of the strain of interest (*lacZ*+) and the appropriate control strain (Δ*lacZ*). Mice were infected intragastrically with approximately ∼10^5^ CFU and sacrificed 24 hours post-infection. Small intestines were homogenized in 1 ml LB+16% glycerol, diluted in LB broth, and plated on LB agar plates containing 100 µg/ml Sm and 40 µg/ml 5-bromo-4-chloro-3-indolyl-β-d-galactopyranoside (X-gal). The competitive index was calculated as the ratio of the mutant compared to the control strain normalized to the input ratio. *In vitro* controls were included in each of these experiments in which the same inoculum was diluted 1∶100 into at least five independent LB cultures and the output ratios of mutant to the control strain were determined on Sm X-gal agar plates as above.

### Polymyxin B killing assays

Polymyxin B killing assays were done as previously described with minor modifications [57]. Briefly, overnight cultures were subcultured 1∶100 into LB and grown at 37°C to the desired OD (OD_600_ = 0.15 and OD_600_ = 0.5). 5 µl polymyxin B (Invitrogen) at 500 µg/ml was added to 45 µl of the above culture in a well of a 96-well polypropylene microtiter plate to obtain a final test concentration of 50 µg/ml polymyxin B. After three hours of incubation at 37°C with shaking, serial dilutions of each culture were plated on LB Sm plates. The percent survival was calculated as (CFU_(polymyxin treatment)_/CFU_(untreated)_)×100. The average percent survival was determined from two biological replicates, each having been done in technical duplicate.

### Evaluation of phage resistance during growth on chitin

Overnight cultures of wild type *V. cholerae* E7946 were serially diluted in filter sterilized pond water to approximately 10^5^ CFU/ml. 10 µl of diluted culture (10^3^ CFU) was used to inoculate 1 ml chitin solution (1% chitin from crab shells [Sigma] in filter sterilized pond water). To assess the impact of phage on the appearance of phase variants under these conditions, approximately 10 PFU of ICP1 was immediately added following inoculation of bacteria (MOI = 0.01). The mixture was allowed to incubate for 24 hours at 30°C statically at which time the mixture was vortexed and plated for CFU. ICP1 was enumerated by adding chloroform to a 100 µl aliquot of the above solution, diluted and plated for PFU with E7946 using the soft agar overlay method as described above.

### Evaluation of the presence of phase variants in cholera stool samples

Three cholera stool samples collected at the ICDDR,B in 2001 and stored in the presence of glycerol were assayed for the presence of isolates with altered O1 antigen. Single isolates from each sample were found to be O1 Inaba that were sensitive to ICP1. wbeL* and manA* mutants of this clinical O1 Inaba isolate were recovered after plating with ICP1 and used to assess the applicability of replica plating on polymyxin B as a tool to identify heterogeneity within a stool sample. Both the wbeL* and manA* isolates in this background failed to grow on LB agar plates containing 800 µg/ml polymyxin B, while the parental strain maintained its ability to grow. Each stool sample was plated on LB agar containing 100 µg/ml Sm and incubated overnight at 37°C. Plates were then replica plated onto polymyxin B plates and incubated overnight at 37°C to identify polymyxin B sensitive isolates in the stool sample. Approximately 5000 colonies were analyzed per stool sample.

### Ethics statement

All animal experiments were done in accordance with NIH guidelines, the Animal Welfare Act and US federal law. The experimental protocol using animals was approved by Tuft University School of Medicine's Institutional Animal Care and Use Committee. All animals were housed in a centralized and AAALAC-accredited research animal facility that is fully staffed with trained husbandry, technical, and veterinary personnel.

## Supporting Information

Figure S1 **Chemical structure of the** ***V. cholerae*** **O1 antigen, serotypes Ogawa (R = CH_3_) and Inaba (R = H).** The O1 antigen is composed of 12–18 repeating units (n) of α(1,2)-linked d-perosamine residues, the amino groups of which are acylated with tetronate.
(TIF)

Figure S2 **The coding sequence of wild type** ***wbeL*** **.** The A_8_ tract which is mutated to A_7_ in wbeL* is indicated below the arrow. The premature stop codon resulting from the frameshift mutation is indicated in red. The putative ATP-binding domain [14] is highlighted in gray.
(TIF)

Figure S3 **The coding sequence of wild type** ***manA*** **.** The A_9_ tracts subject to slipped-strand mispairing are indicated below the arrows. The premature stop codons resulting from the respective frameshift mutations are indicated in red. The conserved PMI motif [20] is highlighted in gray. In addition, amino acids predicted to be involved in zinc ligand binding [78] are indicated with an asterisk (GLN^104^, HIS^106^, GLU^141^, HIS^264^).
(TIF)
