# Supplementary figures and images for: Phase Variable O Antigen Biosynthetic Genes Control Expression of the Major Protective Antigen and Bacteriophage Receptor in Vibrio cholerae O1
Source: PLoS Pathog. 2012 Sep 13;8(9):e1002917. doi: 10.1371/journal.ppat.1002917 (PMC3441752; doi:10.1371/journal.ppat.1002917)

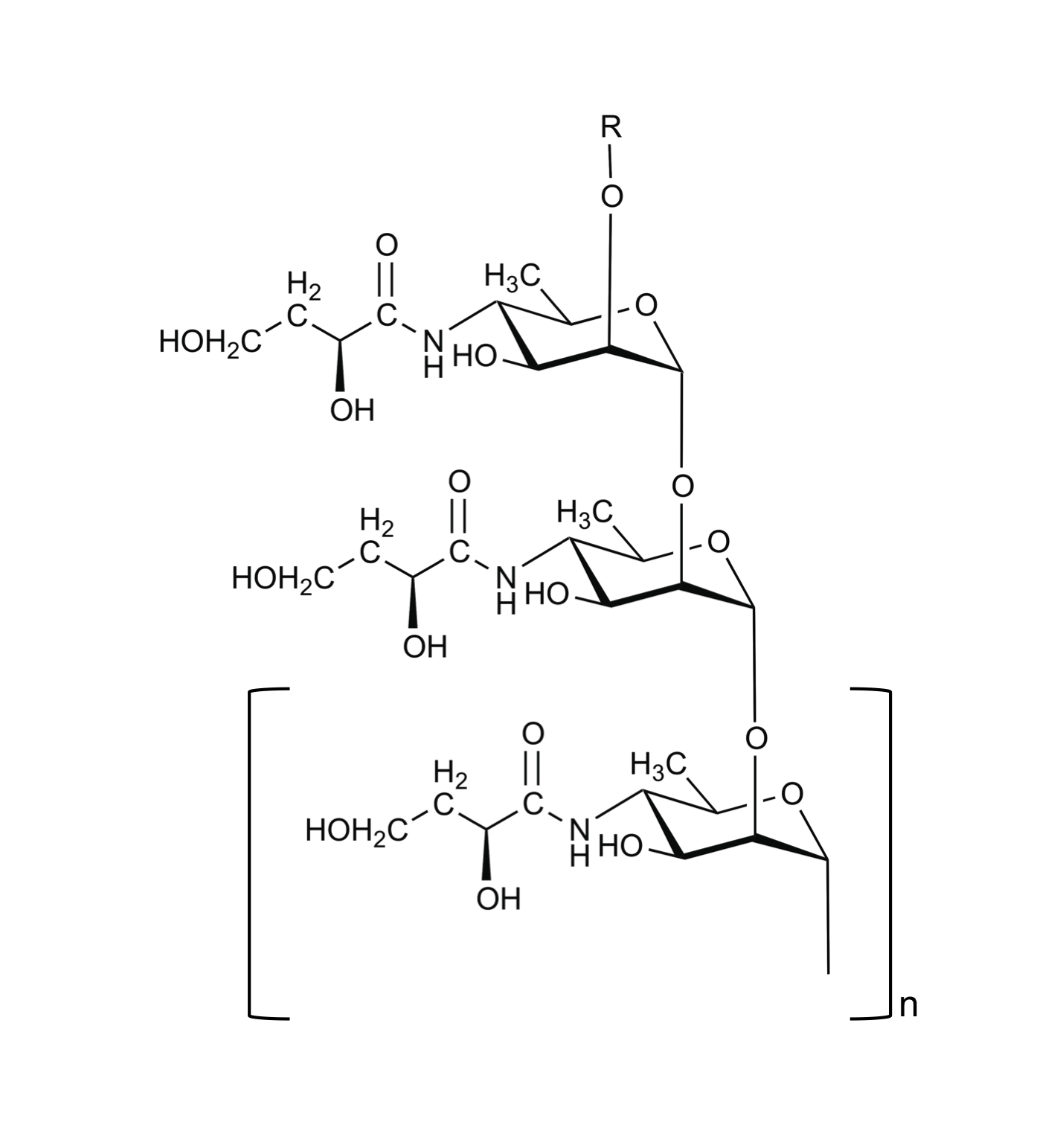

Supplement: Figure S1 — Chemical structure of the V. cholerae O1 antigen, serotypes Ogawa (R = CH3) and Inaba (R = H). The O1 antigen is composed of 12–18 repeating units (n) of α(1,2)-linked d-perosamine residues, the amino groups of which are acylated with tetronate. (TIF) [file ppat.1002917.s001.tif]

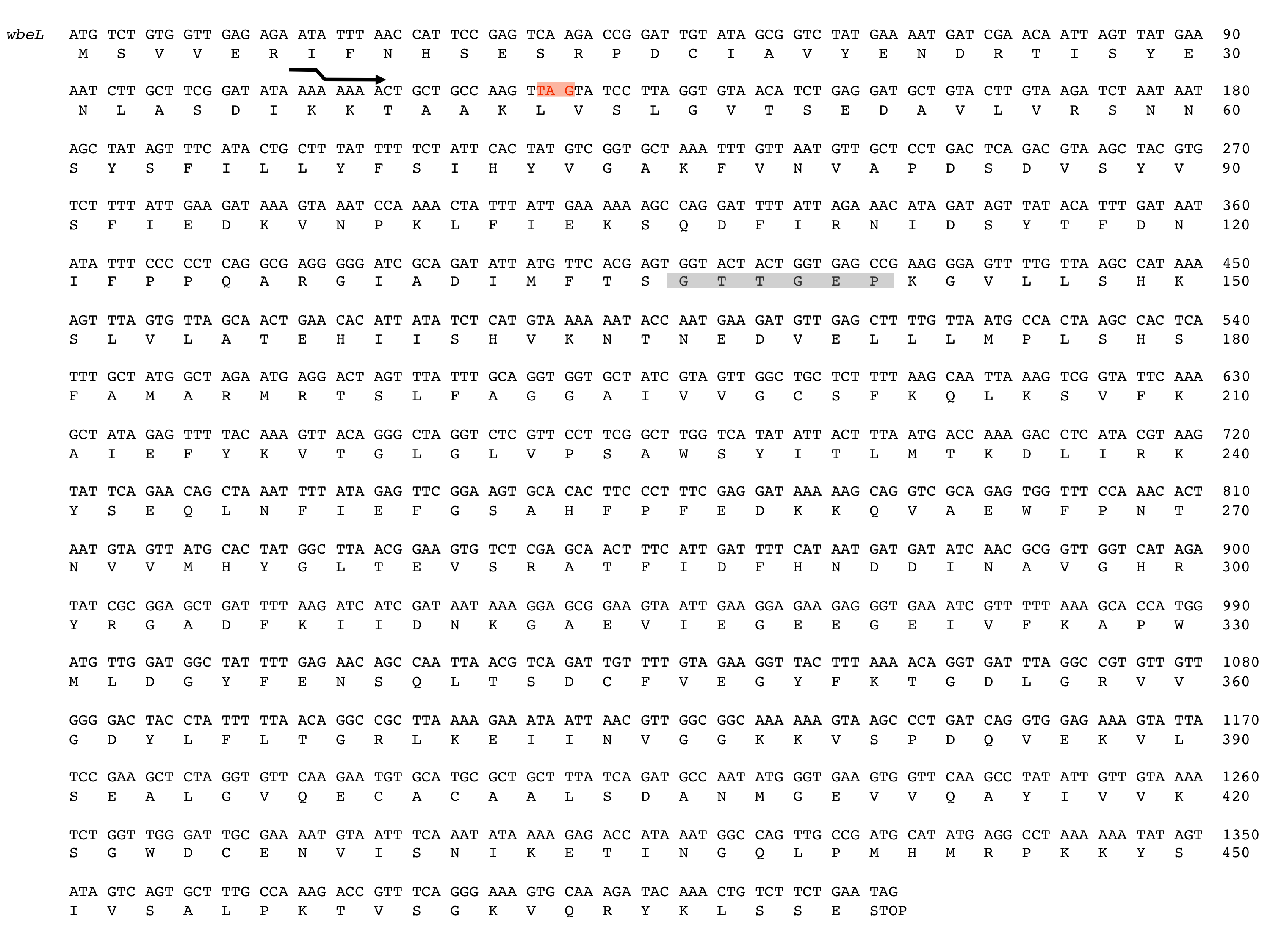

Supplement: Figure S2 — The coding sequence of wild type wbeL . The A8 tract which is mutated to A7 in wbeL* is indicated below the arrow. The premature stop codon resulting from the frameshift mutation is indicated in red. The putative ATP-binding domain [14] is highlighted in gray. (TIF) [file ppat.1002917.s002.tif]

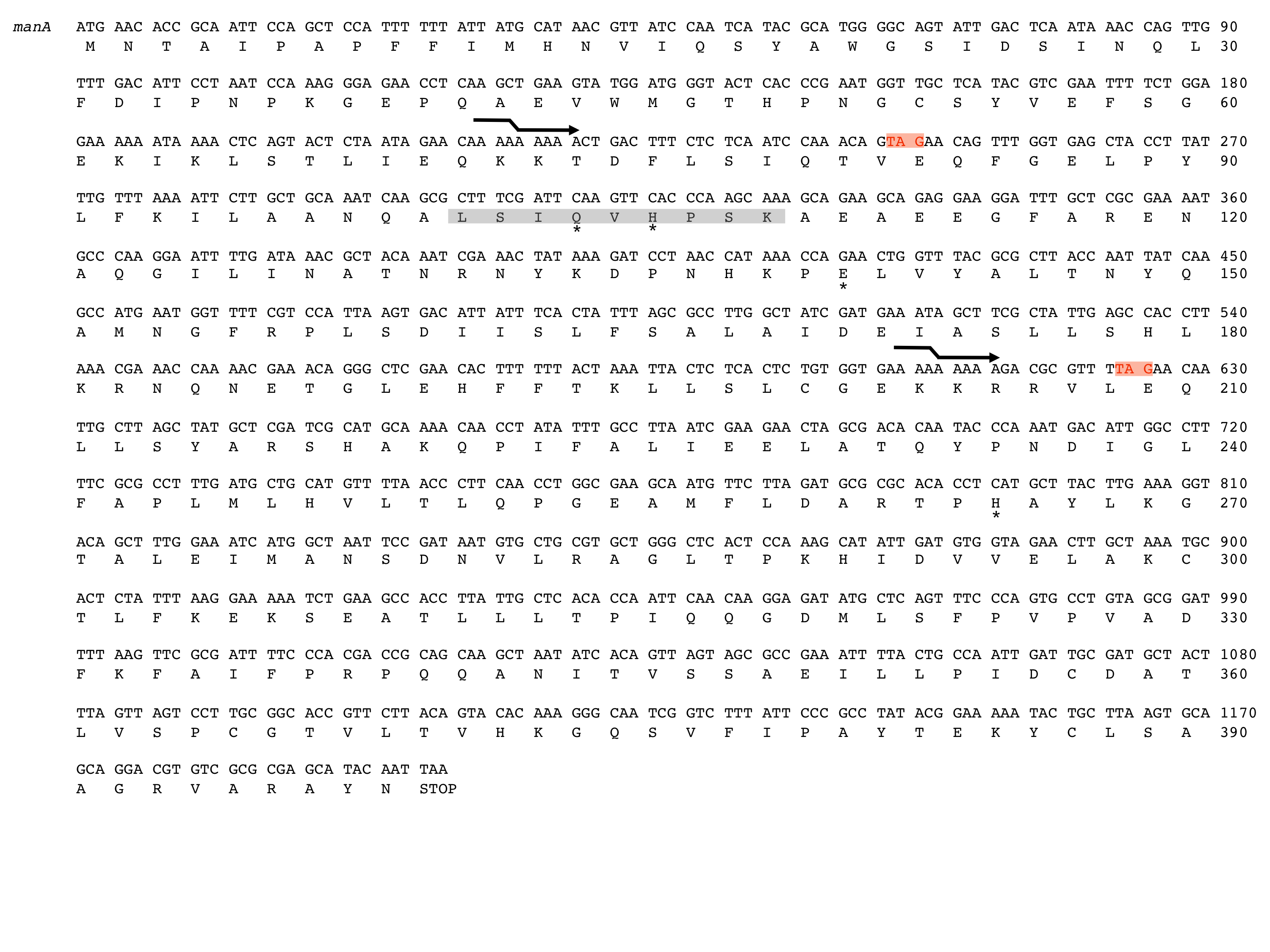

Supplement: Figure S3 — The coding sequence of wild type manA . The A9 tracts subject to slipped-strand mispairing are indicated below the arrows. The premature stop codons resulting from the respective frameshift mutations are indicated in red. The conserved PMI motif [20] is highlighted in gray. In addition, amino acids predicted to be involved in zinc ligand binding [78] are indicated with an asterisk (GLN104, HIS106, GLU141, HIS264). (TIF) [file ppat.1002917.s003.tif]
